# The Ambulatory Teaching Minute: Development of Brief, Case-Based, Evidence-Based Medicine Exercises for the Internal Medicine Resident Continuity Clinic

**DOI:** 10.15766/mep_2374-8265.10909

**Published:** 2020-06-18

**Authors:** Devin Oller

**Affiliations:** 1 Assistant Professor, Division of General Internal Medicine, University of Kentucky College of Medicine

**Keywords:** Ambulatory, Clinic, Internal Medicine, EBM, Evidence-Based Medicine, Case-Based Learning, Primary Care

## Abstract

**Introduction:**

There are limited data on best approaches for evidence-based medicine (EBM) teaching to residents in busy continuity clinic practices. Ambulatory Teaching Minute (ATM) exercises were designed to deliver brief, case-based, faculty-facilitated teaching to residents on high-yield ambulatory EBM topics.

**Methods:**

I developed four ATM exercises, with each one-page handout containing a clinical case, a guided discussion of a research question and study design sparked by the case, a synopsis of a recent research article addressing this question, and a teaching guide for facilitation. Internal medicine residents received these ATM exercises over the course of their monthlong ambulatory block. Surveys that assessed resident engagement were obtained from faculty-facilitators (*N* = 4) and residents (*N* = 6) at the end of the ambulatory block.

**Results:**

Residents were actively engaged in the exercise, with an average engagement score of 3.81 out of 5. Most respondents reported ATM exercises taking 6–10 minutes. The majority of respondents felt ATMs could be realistically completed once per week.

**Discussion:**

In this preliminary assessment of a new tool for EBM teaching in clinic, positive engagement scores among preceptors and residents highlight the potential of ATMs to efficiently and effectively address EBM topics during limited teaching time in clinic.

## Educational Objectives

By the end of this activity, learners will be able to:

1. Develop research questions in PICO format (population, intervention, comparison, outcomes) and outline potential study designs from brief ambulatory-focused clinical vignettes.
2. Interpret and appraise the clinical impact of recent ambulatory-focused studies.

## Introduction

Complex patients, limited time, busy preceptors—the continuity clinic can be a stressful environment for residents.^1^ Given these distractions, it is no surprise that evidence-based medicine (EBM) teaching is limited in the clinic,^2^ despite common ambulatory topics like prevention, women's health, and geriatrics making up almost one-quarter of the American Board of Internal Medicine exam content.^3^ The Accreditation Council for Graduate Medical Education's competency on practice-based learning and improvement requires that residents “appraise and assimilate” scientific evidence to improve their care of patients.^4^ The Ambulatory Teaching Minute (ATM) was developed to address this requirement by making use of prior studies demonstrating that even small blocks of teaching can represent important learning opportunities.^5^ While prior work has shown the positive impact of longitudinal EBM didactics^6,7^ and collaborative EBM sessions,^8^ it is not known how best to deliver EBM teaching in the continuity clinic setting.

ATMs were designed to engage residents in EBM teaching in busy ambulatory academic medicine settings. The one-page ATM handouts consisted of four sections: (1) an ambulatory-focused case description that provided an opportunity to assess and address gaps in clinical knowledge among learners, (2) a guided discussion meant to develop a meaningful research question^9^ and outline the ideal study to answer the question posed by the case, (3) a brief synopsis of a published study that addressed this question, and (4) a brief facilitator-facing discussion guide. The learning objectives of this study were targeted by two unique features of ATMs: First, the ATM format mirrored the natural progression of scientific inquiry, moving participants from clinical case to research question sparked by the case to formulation of a study design, and then allowed learners to compare their imagined study to a real-life one; second, facilitation (through a built-in teaching guide) focused on applicability of the featured study to clinical practice.

In the hectic clinic environment, engaging residents in EBM teaching represents a key initial hurdle to knowledge acquisition. With emerging evidence supporting engagement metrics to assist in developing best practices in facilitation and teaching,^10^ it is critical to focus on learner engagement as new curricula are introduced. Additionally, resident engagement may be associated with long-term knowledge retention.^11^ It is not known how best to engage residents in EBM teaching in the continuity clinic context. In this preliminary work, I assessed the impact of ATMs on resident engagement.

## Methods

Internal medicine residents at the University of Kentucky had monthlong ambulatory blocks in which their continuity clinic sessions were scheduled. They were matched with a continuity clinic preceptor across their entire residency. One to three residents worked with a preceptor during their continuity clinic. There was typically a brief period of time at the start of clinic before patients arrived that could be used for teaching. EBM-focused didactics were integrated into the residency-wide noon conference series and were typically delivered in journal club or traditional lecture formats. ATMs were designed to complement, not replace, the existing curriculum, adding additional content areas not covered by the existing curriculum and centering content in the ambulatory setting (traditionally, EBM didactics at the program focused on inpatient topics). However, no prerequisite EBM knowledge was needed by facilitators or learners for this intervention.

The following protocol was approved by the University of Kentucky Institutional Review Board. I created four ATMs. The articles and topics for these four ATMs were chosen for their timeliness (all articles had been published within the last year) and adaptability to the ATM format (see Appendix A). Each ATM included a synopsis of a recent article pertinent to the case described, as well as optional additional readings.^12-18^ I invited all continuity preceptors for the internal medicine residency program to participate as facilitators, with four preceptors opting in. In an effort to standardize facilitation, these preceptors received an instructional video reviewing the ATM format (see Appendix B for the PowerPoint slides reviewed in the video) 2 weeks prior to their first ATM sessions. All preceptees for the preceptors who opted in were given four ATMs over the course of their ambulatory month. Facilitators were instructed to print out copies of the ATMs for their preceptees; no audiovisual equipment or supplementary supplies were needed for facilitation. In the week prior to completing their ambulatory month, these residents and their preceptors were emailed an invitation to participate in the optional survey to measure resident engagement (see Appendix C). I then analyzed the deidentified survey data. To facilitate development of future ATMs at other sites, I created an ATM development guide and handout template (see Appendix D).

I used a modified version of the previously validated STROBE instrument^19^ to assess learner engagement with the novel teaching format. Total STROBE scores were calculated by adding together the Likert-scale integer associated with each response (1–5), reversing scores for questions 2 and 4. A group mean STROBE score was calculated and divided by nine (the number of questions in the survey) to give an average engagement score from 1 to 5, where 5 demonstrated maximum engagement. An average engagement score greater than 3 was interpreted as more engaged than disengaged. In addition to the engagement instrument, to guide future implementation, both residents and preceptors were asked how long the ATM exercise took to complete and how frequently they would like the ATMs to occur.

## Results

Per preceptor reports, four preceptors served as facilitators of ATM exercises to 24 residents over the course of 3 months, delivering four ATMs to each of these learners. Surveys to measure resident engagement were completed by all four preceptors and six residents (100% and 25% response rates, respectively). The results of the surveys are reviewed in the Table.

**Table.**
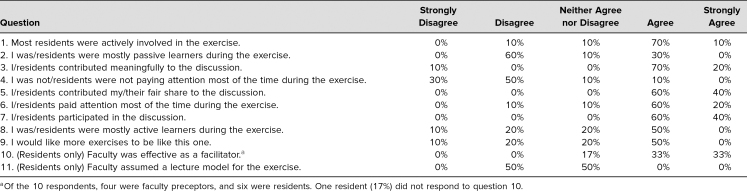
Engagement Survey Responses (*N* = 10)

Using the Fisher exact test (threshold for significance: *p* < .05), I found no significant differences between resident and attending positive responses (agree or strongly agree) versus negative or neutral responses to questions 1–9. Therefore, for the purposes of presenting the data, responses from facilitators and learners are combined. For questions with a negative stem (questions 2 and 4), responses of strongly disagree and disagree were counted as positive.

The group mean STROBE score was 34.3 (range of individual scores: 28–41, *SD* = 4.22). This corresponded to an average engagement score of 3.81 (out of a maximum of 5).

Sixty percent of respondents reported the exercise taking 6–10 minutes, with the next most frequent response (20% of respondents) being 2–5 minutes. In response to the question “How frequently would it be realistic for you to complete an ATM exercise?”, 60% responded once per week, 30% once per month, and 10% twice per week.

## Discussion

To address the challenge of delivering effective EBM teaching in busy ambulatory resident continuity clinics, I developed the ATM exercises and assessed resident engagement during these exercises. ATMs actively engaged residents, as reflected in an average engagement score of 3.81 (maximum score = 5). The ATM was designed to be a collaborative learning exercise, and high scores for resident contribution (questions 3 and 5), participation (question 7), and active learning (question 6) point to the potential benefit of ATMs in team-based environments. While the exercises took slightly longer than expected—a majority of respondents noted that 6–10 minutes were required to complete ATMs—this remained a small time commitment for most, reflected in the fact that 60% of residents and preceptors felt that ATMs could be realistically delivered once per week.

Several lessons learned during the development and implementation of the ATM should be highlighted to inform others interested in using ATMs. First, while facilitation may take less than 10 minutes on average, crafting each of the ATM handouts—from literature search to case creation to facilitator guide assembly and formatting—took approximately 1 hour. Narrowing the scope of the ATM to either a clinical pearl or a key EBM takeaway would likely lower ATM development and facilitation time. Although this more limited focus is not included in the four ATMs presented here, it is reflected in the ATM development guide in Appendix D.

Even with a video facilitation guide, facilitators still had additional questions or clarifications. The main themes of these inquiries were (1) time allocation during facilitation of the sections of the ATM and (2) best practices when only one learner was present. Future work could include assessments that ask participants to rank the various features of the ATM they find most helpful. With respect to 1:1 facilitation, implementation of ATMs at other sites can include guidance that facilitators combine small (two or less) preceptee groups to achieve a target group between four and six participants. In addition, to standardize facilitation, periodic group feedback sessions for facilitators could be helpful in formulating best practices for facilitation and coaching new facilitators.

While I initially sought to assess knowledge retention with a follow-up quiz when residents returned to their ambulatory block after 2 months, I anticipated even lower response rates among residents than the 25% response rate seen with the engagement survey and chose not to include this assessment. For future work, individual or group feedback sessions may provide a more reliable method for measuring knowledge acquisition and retention.

Finally, while a video facilitation guide was used to try to standardize facilitation and each ATM had a teaching guide, it was not known what EBM training or prior knowledge preceptors brought to the exercises or how that prior knowledge impacted resident engagement scores. Were the ATM to be studied across multiple sites, extra attention would need to be paid to standardizing facilitation.

The positive engagement scores seen in this preliminary work point to the ATM's potential as an additional tool in the preceptor's toolbox to address clinical and EBM knowledge gaps among residents. A multisite study with pre- and postintervention knowledge assessments is the next step in measuring ATMs’ impact on ambulatory and EBM curricula. As the clinical demands on residents in ambulatory continuity clinic settings are unlikely to diminish in the coming years, ambulatory-focused learning must continue to become more streamlined and reflective of how residents learn. Sharing ATMs and best practices for facilitation can help refine this intervention and better serve preceptors and residents alike.

## Appendices

Ambulatory Teaching Minutes.pdfFacilitation Guide.pptxEngagement Survey.docxATM Development Guide & Template.docx
*All appendices are peer reviewed as integral parts of the Original Publication.*
