# Supplementary material for: The Ambulatory Teaching Minute: Development of Brief, Case-Based, Evidence-Based Medicine Exercises for the Internal Medicine Resident Continuity Clinic
Source: MedEdPORTAL. 2020 Jun 18;16:10909. doi: 10.15766/mep_2374-8265.10909 (PMC7331953; doi:10.15766/mep_2374-8265.10909)
Supplement: Supplementary file 1 — Ambulatory Teaching Minutes.pdfFacilitation Guide.pptxEngagement Survey.docxATM Development Guide & Template.docx [file mep_2374-8265.10909-s001.zip › A. Ambulatory Teaching Minutes.pdf]

Ambulatory Teaching Minute

Exercise 4

The Case

A 78 year old woman with hyperthyroidism and osteoporosis presents to clinic with **right calf pain and swelling**. She is accompanied by her home health aide. She just came back earlier this week from a visit with her grandchildren that involved a 5 hr flight. On exam, she is afebrile, her pulse is 102, blood pressure 132/74, respiratory rate 10, and O2 saturation 96%. Her right calf is swollen, slightly warm to the touch, and tender to palpation. Pulses are symmetric and strong in her bilateral feet. You send her to radiology for a lower extremity ultrasound which shows a non-collapsing peroneal vein. You are considering what anticoagulation to use for this DVT when the home health aide mentions that the patient had been "slowing down" and "feeling weaker" for months.

The Question

- What research question could help address the issue posed by the above case?
- What type of study would you conduct to answer that question? Why?
- What outcomes would be important to you? How about to the patient?

Fold!

The Paper

What they did: Using retrospective claims databases, the researchers found patients with a new diagnosis of VTE (DVT or PE) receiving either warfarin or rivaroxaban, calculated the patients' frailty scores, and then looked to see if patients developed recurrent VTE or major bleeding at 12 months (composite 1° endpoint). Propensity scores were used to try to reduce bias due to baseline confounding variables.

Why they did it: Frailty predicts poorer outcomes for patients with VTE. Data comparing warfarin and rivaroxaban for the treatment of VTE in frail patients is lacking.

What they found: 12% of the 58k+ patients they identified as having VTE were classified as frail. Patients in the rivaroxaban group were less likely to develop recurrent VTE or major bleeding (HR=0.75, CI 0.57-0.98) compared to the warfarin group. However, looking at major bleeding alone, there was not a significant difference between groups.

Knowing this, what would you tell your patient?

Further Reading

Coleman CI, Turpie AG, Bunj TJ et al. Effectiveness and Safety of Rivaroxaban Versus Warfarin in Frail Patients with Venous Thromboembolism. *The American Journal of Medicine* (2018) 131, 933-938

Austin PC. An Introduction to Propensity Score Methods for Reducing the Effects of Confounding in Observational Studies. *Multivariate Behavioral Research*. 2011;46(3):399-424. doi:10.1080/00273171.2011.568786.

Fold!

Teaching Guide

The Case

- This is a case of a provoked DVT in a frail woman. Consider asking: What characteristics do you think about when defining frailty in older patients? A: While the Physical Frailty Phenotype is the most commonly used tool for defining frailty in the literature, the FRAIL scale is a validated, easy-to-remember scale intended for use in primary care (3+=frail): **F**atigue (*Are you fatigued?*) **R**esistance (*Can you climb one flight of stairs?*) **A**mbulation (*Can you walk one block?*) **I**llnesses (>*5*) **L**oss of weight (*>5%).*

The Question

- What are the relative risks/benefits of using rivaroxaban vs warfarin in frail patients with a new diagnosis of DVT?
- While an RCT would minimize the risk of confounders affecting differences between groups, you're likely stuck with doing a retrospective study as the outcomes you are concerned about (recurrent DVT, major bleeding) are relatively rare and likely to happen months to years down the line. Re: outcomes…Patients likely will have similar outcomes.

The Paper

- Ask: How do propensity scores work? A: A propensity score is simply a measure of the probability that a patient will be in a treatment group based on their characteristics (demographics; comorbidities, ie hyperthyroidism) when they enter the study. In observational studies, like this one, the researchers needed to account for the baseline differences between the group of patients on warfarin and those on rivaroxaban. They used a statistical tool called inverse probability of treatment weighting (IPTW) to create synthetic, well-balanced treatment groups from which they could then analyze outcomes.
- It's likely that this frail patient with DVT could benefit from rivaroxaban over warfarin, but a more detailed bleeding risk assessment should be performed.

Ambulatory Teaching Minute

Exercise 1

The Case

A 54 year-old man presents to clinic with one day of **right-sided flank pain and nausea that worsens with urination.** He is afebrile, his blood pressure is 154/82, and on exam he has no rebound or guarding on palpation of the abdomen, but reports exquisite tenderness to palpation of the right costovertebral angle. You are concerned about nephrolithiasis and send him for a CT scan of the Abdomen and Pelvis. The CT shows a 5mm ureteral stone without nephrolithiasis. UA shows 2+ blood, but no leuk esterase. BMP reveals stable SCr of 1.1. You recommend that he maintain good hydration and prescribe a short course of NSAIDs for pain. He asks whether there are any medications available to increase the chance that the stone passes or speed his return to work.

The Question

- What research question could help address the issue posed by the above case?
- What type of study would you conduct to answer that question? <u>Why?</u>
- What outcomes would be important to <u>you</u>? How about to the <u>patient</u>?

Fold!

Fold!

The Paper

<u>What they did</u>: a placebo-controlled, double-blind clinical trial of Tamsulosin in adult patients presenting to the ED with a symptomatic ureteral stone <9mm in diameter (confirmed by CT).

<u>Why they did it</u>: current conservative management guidelines for nephrolithiasis include Tamsulosin (0.4 mg qd) as medical expulsive therapy based off of systematic review, which showed it improved the likelihood of clearance (mostly in larger stones). But 3 large clinical trials have called into question whether the therapy is effective for stones <9mm in diameter.

<u>What they found</u>: No significant difference in stone passage rate at 28 days between Tamsulosin and placebo. No difference in need for surgery. No difference in time to return to work. Rates of ejaculatory dysfunction were higher in the treatment group.

What would you tell your patient?

Further Reading

Meltzer AC, Burrows PK, Wolfson AB et al. Effect of Tamsulosin on Passage of Symptomatic Ureteral Stones: A randomized clinical trial. *JAMA Intern Med.* 2018; 178(8): 1051-1057.

Dahm P, Hollingsworth JM. Medical Expulsive Therapy for Ureteral Stones—Stone Age Medicine. *JAMA Intern Med.* 2018; 178(8): 1058-1059.

Fold!

Fold!

Teaching Guide

The Case

This is a case of symptomatic nephrolithiasis. Consider asking:

Q: What stones can we as primary care doctors handle? What's out of our purview?

A: We need to make sure they have not developed urosepsis (a clinical dx) or obstruction (a dx made with CT or US). If the stone is >10mm, it warrants Urological consultation.

The Question

- What research question could help address the issue posed by the above case? *Is there benefit to prescribing Tamsulosin or another medical expulsive agent to help improve the chance of stone passage*?
- What type of study would you conduct to answer that question? <u>Why?</u> *RCT. Minimizes bias. Important to emphasize: generalizability is dependent on the study population, and in this case, there was a diverse group of participants.*
- What outcomes would be important to <u>you</u>? How about to the <u>patient</u>? *Passage of stone, need for surgery/procedures, time to return to work.* | *For patient: pain, time to return to work, need for surgery/procedures*.

The Paper

Key points to emphasize: 1) These findings are consistent with another recent trial (Pickard et al 2015), 2) 3/4s of the study group had stones less than 4 mm in size, so it's unclear if there's significant benefit for the 4-9mm crowd, 3) a recent study from China showed significant benefit with Tamsulosin for patients with 5-9mm stones. So while there's emerging data that suggest a1-blockers for small stones (<5mm) is probably unnecessary, there could still be benefit for patients with larger stones.

Ambulatory Teaching Minute

Exercise 2

The Case

A 31-year-old transgender female presents to clinic wishing to **discuss initiating hormone therapy with estrogen**. She reports that she is in good health. She sees a psychiatrist for anxiety, which she says is well controlled on an SSRI. She smokes ½-pack of cigarettes per day. She denies any family history of early heart attacks or strokes, nor are there any clotting disorders in her family. She has several friends who take estrogen and she's excited to begin therapy. However, she reports that one of her friends recently developed a clot in her leg, and expresses concern about the risk of clots and other serious cardiovascular side effects with estrogen. Her blood pressure is 122/74 in the clinic. An EKG shows normal sinus rhythm and no signs of ischemia.

The Question

- What research question could help address the issue posed by the above case?
- What type of study would you conduct to answer that question? Why?
- What outcomes would be important to you? How about to the patient?

Fold!

Fold!

The Paper

What they did: Matched transgender members of an integrated health system to cisgender cohorts in the EHR and followed them until they had an acute cardiovascular event (including VTE, ischemic stroke and myocardial infarction), died, or left the health system. Subgroups of patients on hormone therapy were prespecified.

Why they did it: The evidence addressing the issue of cardiovascular risk in transgender patients receiving hormone therapy is limited; what evidence is available is drawn from small studies with very few reported events

What they found: Average follow-up was about 4 years. The adjusted hazard ratio for VTE in transfeminine patients who started estrogen compared to reference men was 3.2 (CI 1.5-6.5). For stroke, HR=2.3 (1.2-4.3). Differences in MI incidence were not significant. VTE rates increased only after 2 years of follow-up and continued to rise for another 5 to 6 years.

Knowing this, what would you tell your patient?

Further Reading

Getuhun D, Nash R, Flanders WD et al. Cross-sex hormones and acute cardiovascular events in transgender persons: a cohort study. *Annals of Int Med.* 2018. 169(4): 205-213.
UCSF Trangender Health Learning Center. http://transhealth.ucsf.edu/

Fold!

Fold!

Teaching Guide

The Case

- Consider asking learners what questions they would ask cis-gendered individuals to ascertain their risk of clot (A: prior VTE, obesity, immobility, OCPs, smoking).
- Consider asking what medications are typically prescribed to transwomen as hormone therapy. (A: Estradiol, typically in patch, oral or depot injection formulae; plus or minus anti-androgen therapy like spironolactone, finasteride).

The Question

- A: What increased risks of serious cardiovascular events are transwomen exposed to with the use of hormone therapy?
- A: Because the events are relatively rare, you need to have a high N, and a long follow-up period, to capture a difference between exposed and unexposed individuals. For that reason, the prospective cohort study makes the most sense. Placebo controlled trials of hormones in transgender individuals are likely not ethical.
- A: For you: MI, CVA, VTE. For patient: probably the same, though would also want to know what % stopped therapy.

The Paper

- Reminder that a hazard ratio is a measure of the effect of an exposure to a group over *time*. The patterns in risk over time in this study were different compared to the existing literature: whereas other smaller studies showed a spike in risk in the first year of therapy, then a plateau, but here patients saw rising risk for another 5-6 years.
- Key point: while these risks are stark, they must be weighed against the benefits of treatment. Hormone therapy for many transgender patients is critical to their physical and emotional wellbeing.

Ambulatory Teaching Minute

Exercise 3

The Case

A 45 year-old Caucasian man with obesity (BMI=31) and diabetes mellitus type II presents to clinic to with **concerns about his heart health**. He is a lifelong non-smoker. His only medication is metformin 1000 mg tabs, which he takes twice a day. He has no history of MI or stroke, but his father died of stroke at age 60. His blood pressure is 132/82 today (134/80 on the nursing visit two weeks prior). Review of his labs show an HgbA1c of 7.2%, Total Cholesterol of 300 mg/dL, HDL cholesterol of 50 mg/dL, and LDL cholesterol of 120 mg/dL. After discussing the risks and benefits of statin therapy, he states that he is not interested in starting a statin, but asks you if taking a baby aspirin could help reduce his risk of heart attack or stroke.

The Question

- What research question could help address the issue posed by the above case?
- What type of study would you conduct to answer that question? Why?
- What outcomes would be important to you? How about to the patient?

FOLD

The Paper

What they did: Randomized over 15,000 patients with T2DM but no prior vascular events to either low-dose aspirin or placebo, and followed them for a mean of 7.4 years to evaluate their risk of developing vascular events, major bleeding events, and gastrointestinal malignancy.

Why they did it: The balance of benefits and risks associated with aspirin use for primary prevention in diabetics is not clear. Retrospective studies previously suggested that aspirin use may be associated with a reduction in GI cancer risk, but these studies were limited in their duration of follow-up.

What they found: Adherence to aspirin and placebo were not significantly different. The Number Needed to Treat (NNT) for the primary efficacy outcome (vascular event) was 91, and 112 to cause a major bleeding event. There was no difference in GI cancer incidence in the treatment vs the placebo arm.

Knowing this, what would you tell your patient?

Further Reading

Bowman L, Mafham M, Collins R et al. Effects of Aspirin for Primary Prevention in Persons with Diabetes Mellitus. *NEJM*. August 2018

McAlister FA. The "number needed to treat" turns 20 — and continues to be used and misused. *CMAJ : Canadian Medical Association Journal*. 2008;179(6):549-553. doi:10.1503/cmaj.080484.

FOLD

Teaching Guide

The Case

- This case concerns aspirin for primary prevention of major vascular events in patients with T2DM. Before jumping into the pros and cons of aspirin, consider asking, "What else would guidelines suggest we do to lower this patient's CVD risk?" (A: BP→he has stage I HTN (130-139/80-89) but ASCVD risk <10%, so only nonpharm therapy is recommended. Cholesterol→his 10-yr ASCVD risk is 7.8%, which would otherwise suggest the need for statin, which he declines. Obesity→ACC guidelines would recommend offering high-intensity lifestyle intervention).

The Question

- Research question: In patients with T2DM and no known cardiovascular disease, how do the potential benefits (lower risk of vascular events) of low-dose aspirin compare to the risks (major bleeding events)?
- Remind learners: Because of the low event rate, you know you will need a large N and a long follow-up period. An RCT will minimize bias by evenly distributing confounders.
- You: MI, CVA, TIA, GI cancer risk, major bleeding event. Patient: MI and CVA > TIA. GI cancer risk. Major and minor bleeding events.

The Paper

- Ask: Would you prespecify any subgroups within this population before collecting data? Why? The study did pre-define subgroups by their CVD risk upon entry into low (<5% 5 year major vasc event risk), medium (5-10%, or high (>10%). This makes sense—consider that cholesterol and BP guidelines are now tied to ASCVD risk, so by creating these groups you may capture a subgroup with greater benefit:risk ratio. (They unfortunately did not find a difference by subgroup)
- Ask: What is a good NNT (Reminder: NNT = 1/ARR) in this case? A: It depends (on baseline risk, time frame, outcomes)!
