# Supplementary material for: The Ambulatory Teaching Minute: Development of Brief, Case-Based, Evidence-Based Medicine Exercises for the Internal Medicine Resident Continuity Clinic
Source: MedEdPORTAL. 2020 Jun 18;16:10909. doi: 10.15766/mep_2374-8265.10909 (PMC7331953; doi:10.15766/mep_2374-8265.10909)
Supplement: Supplementary file 1 — Ambulatory Teaching Minutes.pdfFacilitation Guide.pptxEngagement Survey.docxATM Development Guide & Template.docx [file mep_2374-8265.10909-s001.zip › B. Facilitation Guide.pptx]

## Slide 1
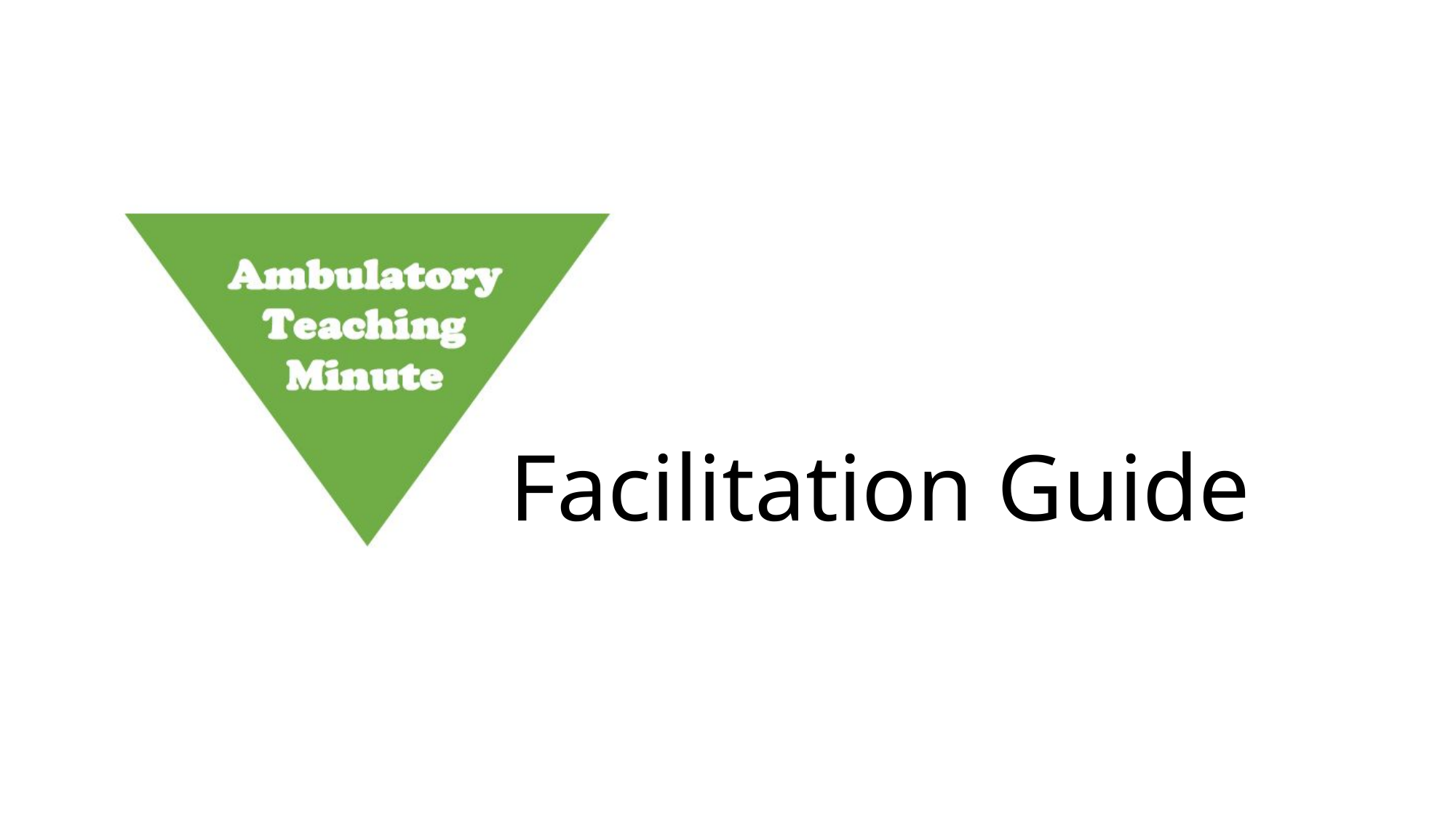

# Facilitation Guide

## Slide 2
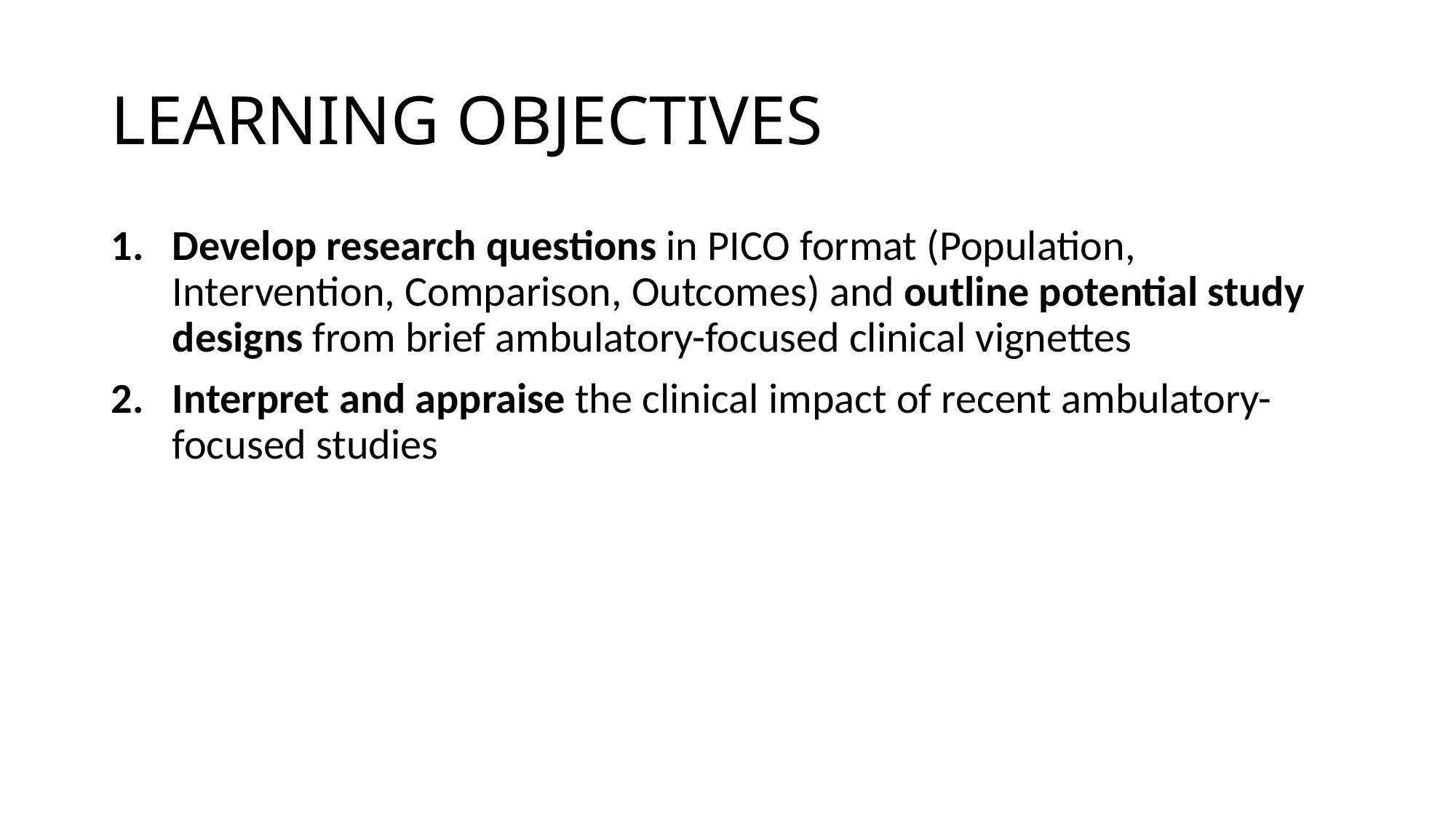

# LEARNING OBJECTIVES
Develop research questions in PICO format (Population, Intervention, Comparison, Outcomes) and outline potential study designs from brief ambulatory-focused clinical vignettes
Interpret and appraise the clinical impact of recent ambulatory-focused studies

## Slide 3
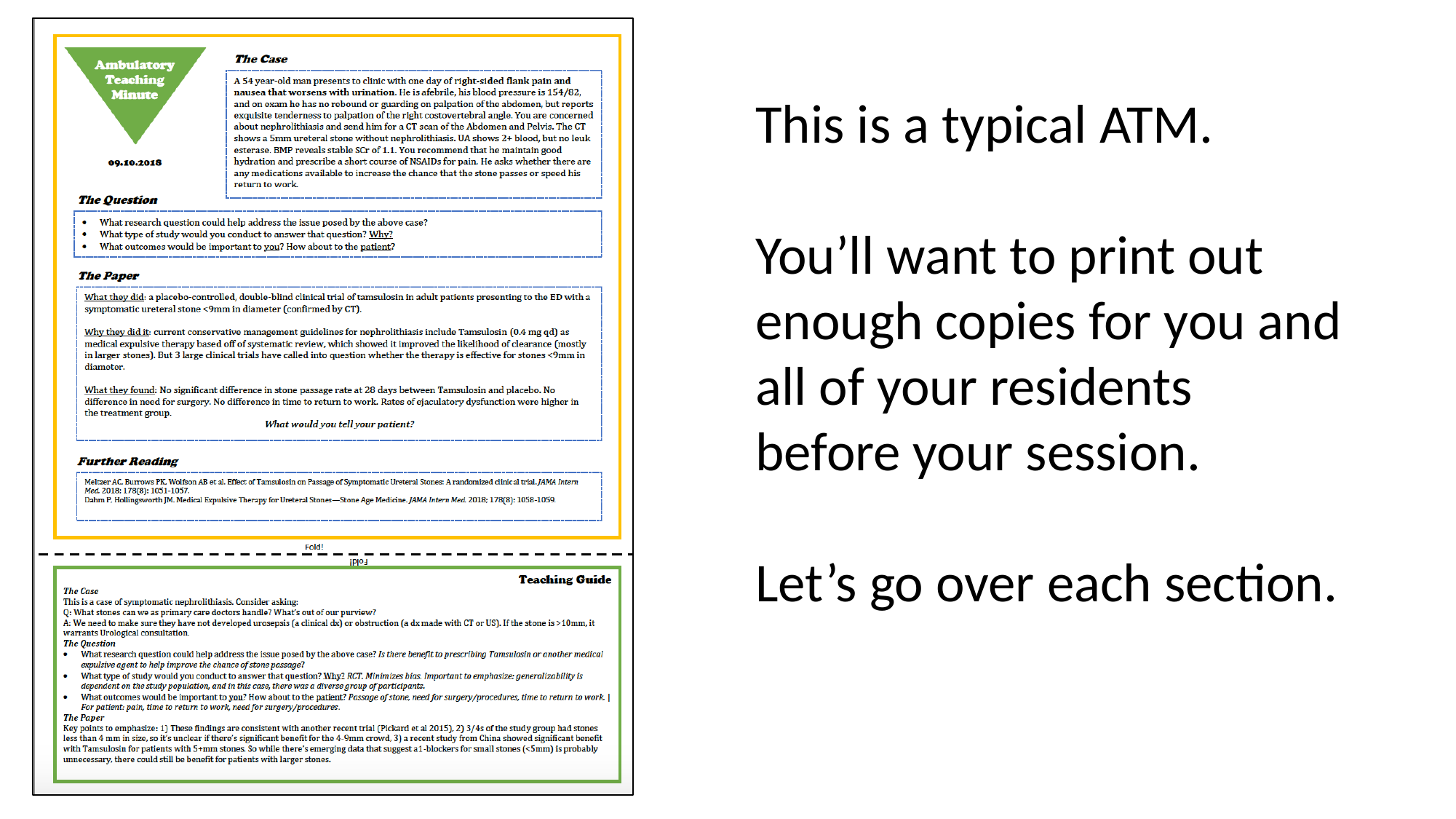

This is a typical ATM.
You’ll want to print out enough copies for you and all of your residents before your session.
Let’s go over each section.

## Slide 4
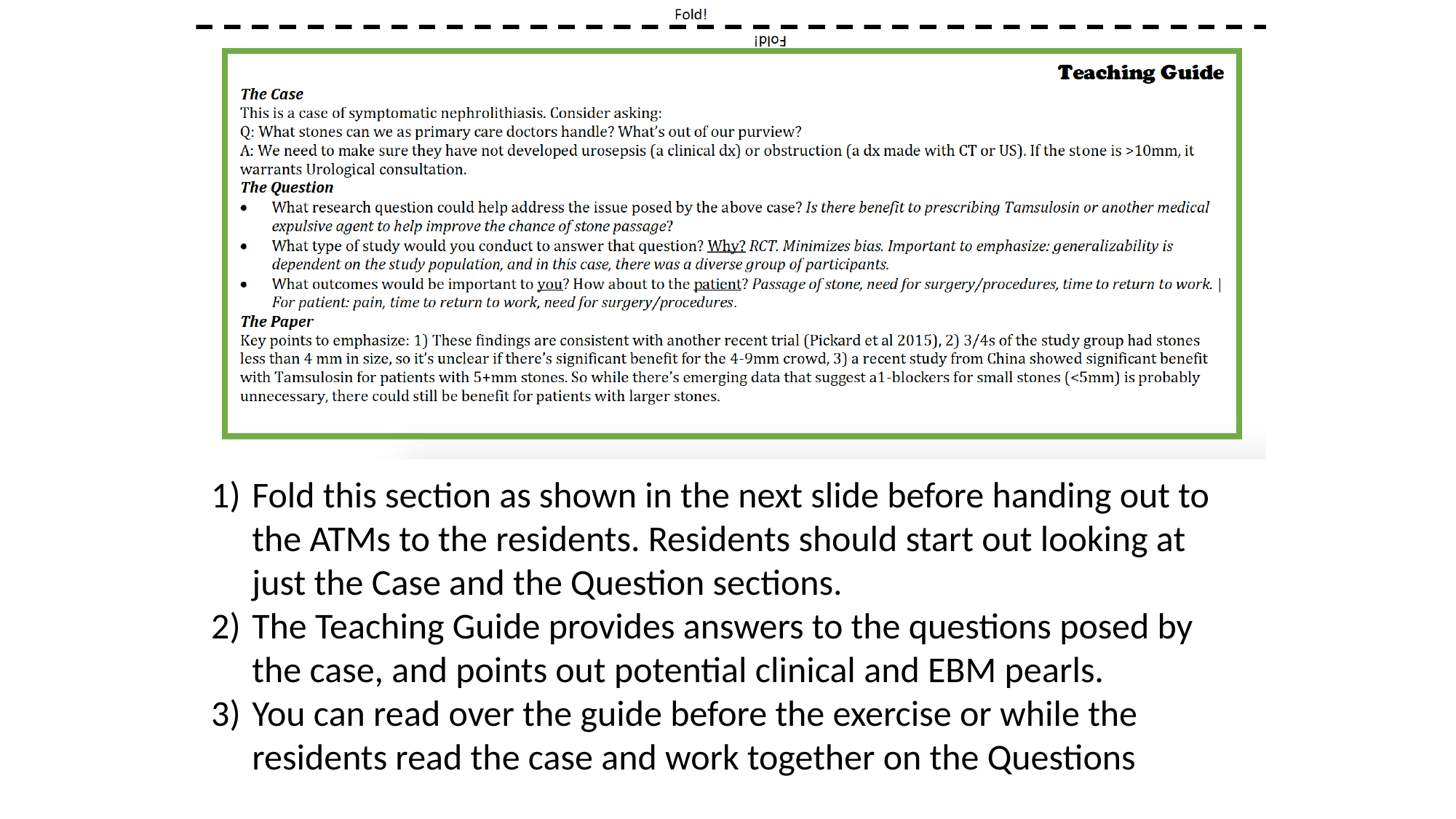

Fold this section as shown in the next slide before handing out to the ATMs to the residents. Residents should start out looking at just the Case and the Question sections.
The Teaching Guide provides answers to the questions posed by the case, and points out potential clinical and EBM pearls.
You can read over the guide before the exercise or while the residents read the case and work together on the Questions

## Slide 5
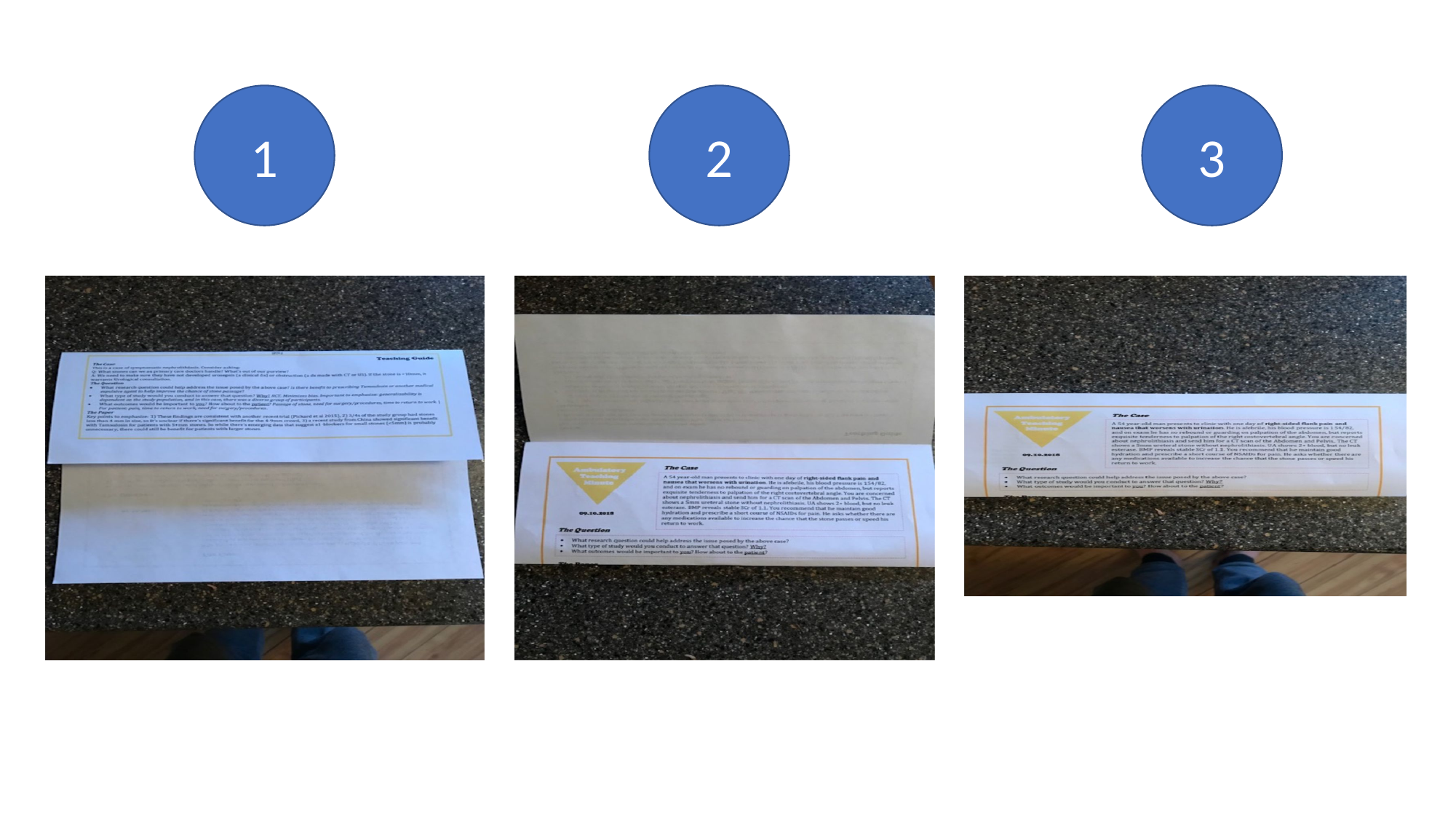

1
2
3

## Slide 6
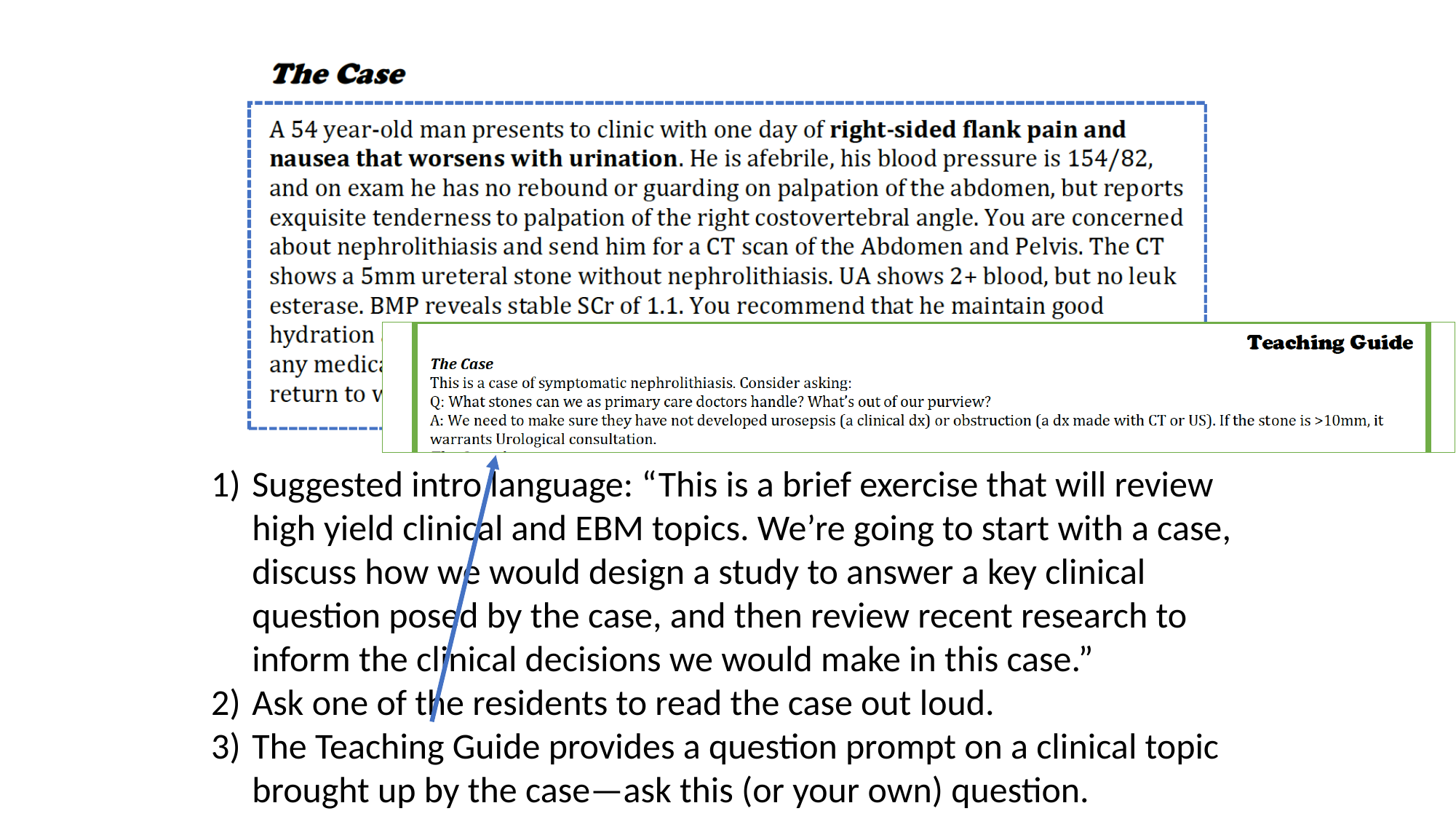

Suggested intro language: “This is a brief exercise that will review high yield clinical and EBM topics. We’re going to start with a case, discuss how we would design a study to answer a key clinical question posed by the case, and then review recent research to inform the clinical decisions we would make in this case.”
Ask one of the residents to read the case out loud.
The Teaching Guide provides a question prompt on a clinical topic brought up by the case—ask this (or your own) question.

## Slide 7
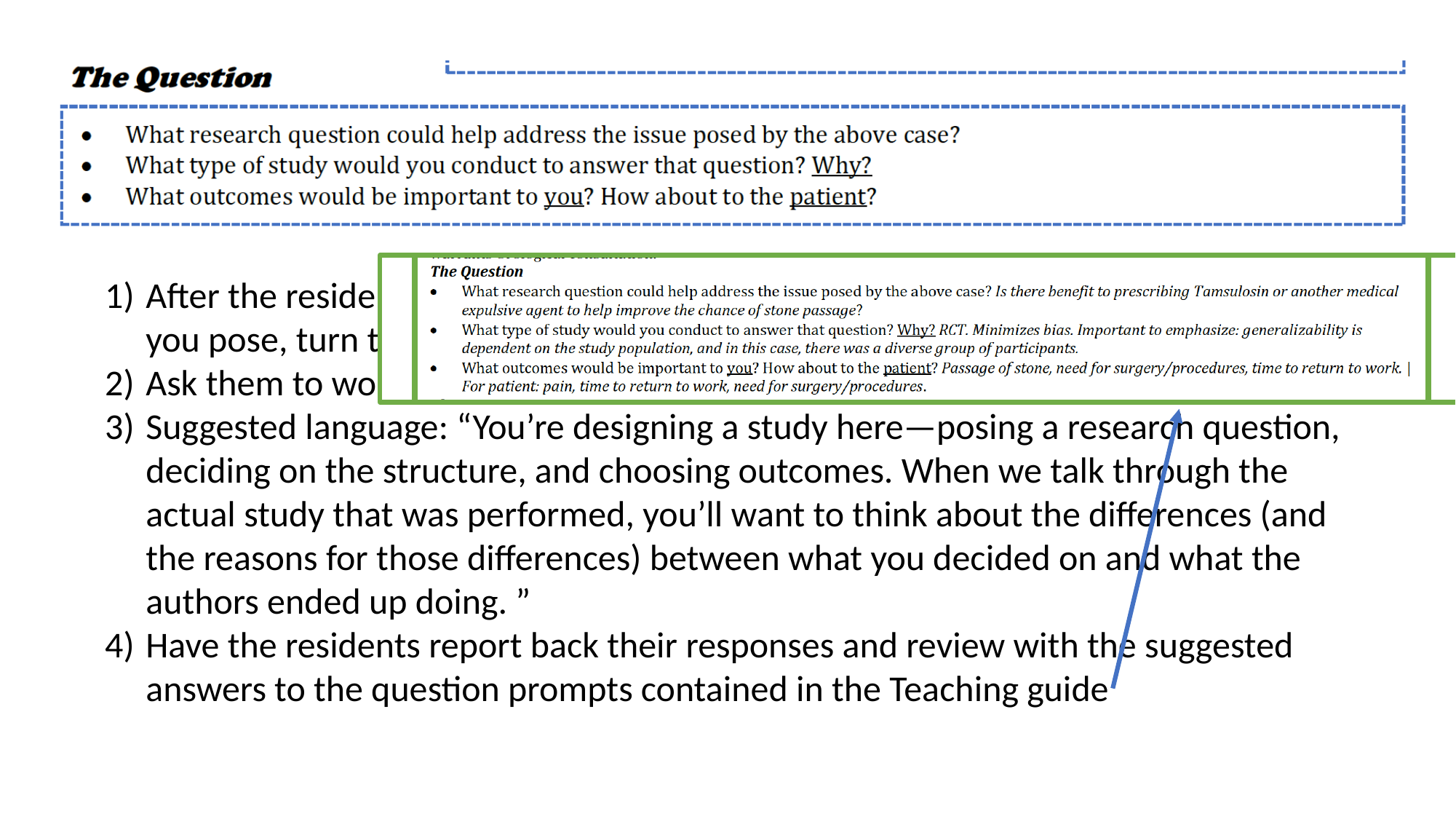

After the residents finish reading the case and answering the clinical question(s) you pose, turn their attention to the next section on the ATM—”The Question”.
Ask them to work in groups of 2-3 to respond to the three question prompts.
Suggested language: “You’re designing a study here—posing a research question, deciding on the structure, and choosing outcomes. When we talk through the actual study that was performed, you’ll want to think about the differences (and the reasons for those differences) between what you decided on and what the authors ended up doing. ”
Have the residents report back their responses and review with the suggested answers to the question prompts contained in the Teaching guide

## Slide 8
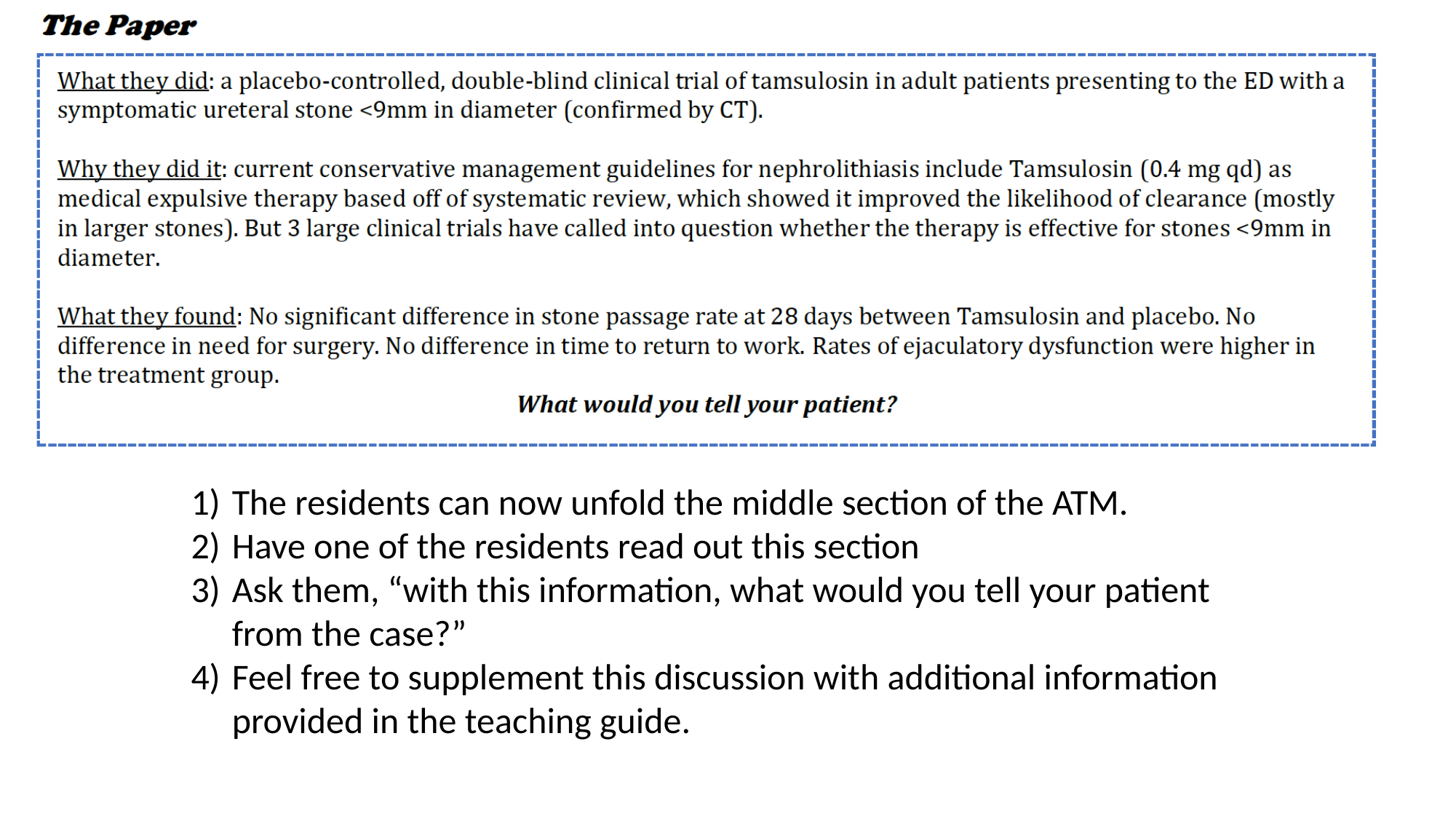

The residents can now unfold the middle section of the ATM.
Have one of the residents read out this section
Ask them, “with this information, what would you tell your patient from the case?”
Feel free to supplement this discussion with additional information provided in the teaching guide.

## Slide 9
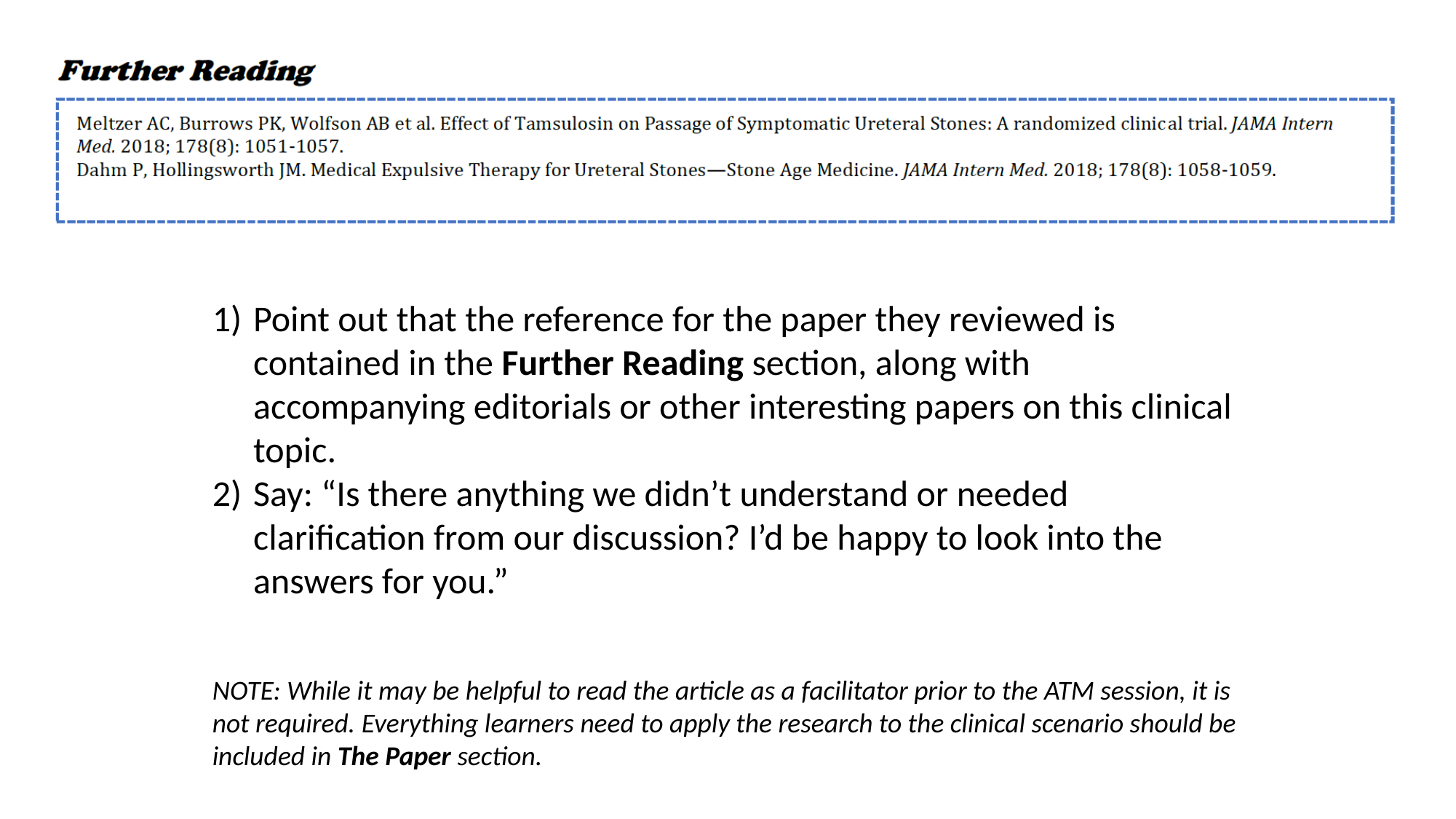

Point out that the reference for the paper they reviewed is contained in the Further Reading section, along with accompanying editorials or other interesting papers on this clinical topic.
Say: “Is there anything we didn’t understand or needed clarification from our discussion? I’d be happy to look into the answers for you.”
NOTE: While it may be helpful to read the article as a facilitator prior to the ATM session, it is not required. Everything learners need to apply the research to the clinical scenario should be included in The Paper section.
