# Supplementary material for: The Ambulatory Teaching Minute: Development of Brief, Case-Based, Evidence-Based Medicine Exercises for the Internal Medicine Resident Continuity Clinic
Source: MedEdPORTAL. 2020 Jun 18;16:10909. doi: 10.15766/mep_2374-8265.10909 (PMC7331953; doi:10.15766/mep_2374-8265.10909)
Supplement: Supplementary file 1 — Ambulatory Teaching Minutes.pdfFacilitation Guide.pptxEngagement Survey.docxATM Development Guide & Template.docx [file mep_2374-8265.10909-s001.zip › C. Engagement Survey.docx]

**Appendix C – Engagement Survey**

*****Note: residents to complete all questions. Faculty to complete questions 1-9

| **Question** | **Strongly Disagree** | **Disagree** | **Neither Agree nor Disagree** | **Agree** | **Strongly Agree** |
| --- | --- | --- | --- | --- | --- |
| 1. Most residents were actively involved in the exercise | 1 | 2 | 3 | 4 | 5 |
| 1. I was/residents were mostly passive learners during the exercise | 1 | 2 | 3 | 4 | 5 |
| 1. I/residents contributed meaningfully to the discussion | 1 | 2 | 3 | 4 | 5 |
| 1. I was not/residents were not paying attention most of the time during the exercise | 1 | 2 | 3 | 4 | 5 |
| 1. I/residents contributed my/their fair share to the discussion | 1 | 2 | 3 | 4 | 5 |
| 1. I/residents paid attention most of the time during the exercise | 1 | 2 | 3 | 4 | 5 |
| 1. I/residents participated in the discussion | 1 | 2 | 3 | 4 | 5 |
| 1. I was/residents were mostly active learners during the exercise | 1 | 2 | 3 | 4 | 5 |
| 1. I would like more exercises to be like this one | 1 | 2 | 3 | 4 | 5 |
| 1. Faculty was effective as a facilitator | 1 | 2 | 3 | 4 | 5 |
| 1. Faculty assumed a lecture model for the exercise | 1 | 2 | 3 | 4 | 5 |

The above questions were adapted from a survey used for students to self-assess engagement in classroom activities adapted from the STROBE instrument. (O'Malley et al. Validation of an observation instrument for measuring student engagement in health professions settings. *Eval Health Prof*. 2003;26(1):86–103)

**Additional Questions**

Answered by all

| **Question** |
| --- |
| 1. How long did it take for you and your preceptor/preceptee to complete the ATM?   Less than 2 minutes 2-5 minutes 6-10 minutes More than 10 minutes |
| 1. How frequently would it be realistic for you to complete an ATM with your preceptor/preceptee?   1 per ambulatory block 1 per week 2 Per week 3+ per week |
