# Supplementary material for: The Ambulatory Teaching Minute: Development of Brief, Case-Based, Evidence-Based Medicine Exercises for the Internal Medicine Resident Continuity Clinic
Source: MedEdPORTAL. 2020 Jun 18;16:10909. doi: 10.15766/mep_2374-8265.10909 (PMC7331953; doi:10.15766/mep_2374-8265.10909)
Supplement: Supplementary file 1 — Ambulatory Teaching Minutes.pdfFacilitation Guide.pptxEngagement Survey.docxATM Development Guide & Template.docx [file mep_2374-8265.10909-s001.zip › D. ATM Development Guide & Template.docx]

**Appendix – Creating an Ambulatory Teaching Minute**

*The following is a step-by-step guide to creating your own ATM. The ATM Template follows on page 2.*

| **1** | **Find your article & Write “The Paper” Section**  Many journals have “Journal Club” or similar features that provide synopses of recent high-yield literature. This is a good place to start. Articles that already have editorials/comments written may have additional points you can adapt to the ATM.  “The Paper” section provides learners with a brief synopsis of 1) the overall approach the authors took (“What they did”), 2) background on the clinical question they were trying to answer (“Why they did it”), and 3) key findings and summary statistics from the paper (“What they found”) . Remember: the article is NOT being read in full by anyone during the exercise, so this synopsis should include everything learners need to interpret how the research can be applied to the clinical scenario. |
| --- | --- |
| **2** | **Pick your Focus & Write “The Takeaway” Section**  Some articles lend themselves to a clinical focus—“Would this change *my* practice?” can be a good question to steer you towards emphasizing the clinical take-aways. Other articles highlight important principles in EBM. To maximize teaching efficiency, we recommend choosing one or the other. “The Takeaway” should be a pithy sentence or two that summarizes the key clinical or EBM teaching point. |
| **3** | **Write the Case**  Your imaginary patient should match the inclusion/exclusion criteria of the study, and the case should include enough data points for learners to quickly pick up on the research question posed by the case. Having your study already chosen and a clear idea of the ATM’s takeaways will help you focus the case and minimize distracting data points. |
| **4** | **Summarize key points in the Teaching Guide**  At this point in ATM development, you should already have your research question (the second item on the teaching guide) and the takeaway (the last item on the teaching guide).  The Case section in the teaching guide should give pointers to facilitators to help steer learners towards the right research question or highlight a key clinical teaching point.  The Paper section in the teaching guide should complement the synopsis given to learners—additional summary statistics, shortcomings of the study pointed out in the discussion, and definitions of key EBM terms are helpful here. |

**Fold**

**Fold**

*The Takeaway*

mm.dd.yy

Teaching Guide

***The Case***

Text

***The Question***

Text

***The Paper***

Text

***The Takeaway***

Clinical:

(Or)

EBM Basics:

Author. Title. *Journal* dd/mm/yy

What they did: text

Why they did it: text

What they found: text

***Knowing this, what would you tell your patient?***

- What research question could help address the issue posed by the above case?
- What type of study would you conduct to answer that question? Why?
- What outcomes would be important to you? How about to the patient?

*The Paper*

*The Question*

Text

*The Case*

**Ambulatory**

**Teaching**

**Minute**
